# Bone-to-Bone and Implant-to-Bone Impingement: A Novel Graphical Representation for Hip Replacement Planning

**DOI:** 10.1007/s10439-020-02451-x

**Published:** 2020-01-22

**Authors:** Arnab Palit, Richard King, Zoe Hart, Yolanda Gu, James Pierrepont, Mark T. Elliott, Mark A. Williams

**Affiliations:** 1 WMG, The University of Warwick, Coventry, CV4 7AL UK; 2 Department of Trauma & Orthopaedics, University Hospitals Coventry and Warwickshire NHS Trust, Coventry, UK; 3 Optimized Ortho, 17 Bridge Street, Pymble, NSW 2073 Australia; 4 Corin Ltd, Corinium Centre, Cirencester, Gloucestershire GL7 1YJ UK

**Keywords:** Total hip replacement, Prosthetic impingement, Bony impingement, Implant orientation, Hip joint, Activities of daily living

## Abstract

Bone-to-bone impingement (BTBI) and implant-to-bone impingement (ITBI) risk assessment is generally performed intra-operatively by surgeons, which is entirely subjective and qualitative, and therefore, lead to sub-optimal results and recurrent dislocation in some cases. Therefore, a method was developed for identifying subject-specific BTBI and ITBI, and subsequently, visualising the impingement area on native bone anatomy to highlight where prominent bone should be resected. Activity definitions and subject-specific bone geometries, with planned implants were used as inputs for the method. The ITBI and BTBI boundary and area were automatically identified using ray intersection and region growing algorithm respectively to retain the same ‘conical clearance angle’ obtained to avoid prosthetic impingement (PI). The ITBI and BTBI area was then presented with different colours to highlight the risk of impingement, and importance of resection. A clinical study with five patients after 2 years of THA was performed to validate the method. The results supported the study hypothesis, in that the predicted highest risk area (red coloured zone) was completely/majorly resected during the surgery. Therefore, this method could potentially be used to examine the effect of different pre-operative plans and hip motions on BTBI, ITBI, and PI, and to guide bony resection during THA surgery.

## Introduction

Total Hip Arthroplasty (THA) produces excellent intermediate to long-term results in accomplishing the primary objectives of enabling patients to reinstate their activities of daily living (ADLs) without pain or restriction.^2^,^5^,^7^ However, there are still many post-operative complications associated with THA, with aseptic loosening and dislocation being two of the most common.^30^,^33^ Although the overall dislocation rate has decreased over the past two decades,^15^,^26^ a significant number of patients continue to experience recurrent episodes. Such recurrent dislocations, defined as two or more occurrences,^10^ occurred in over 60% of patients at a minimum follow-up of 1 year after the first dislocation^10^,^26^ and over 50% of these patients required revision surgery.^10^,^26^ It was reported that impingement is the major cause of restricted range of motion (ROM) and post-THA dislocation.^16^,^28^ The risk factors that are associated with impingement include design of implants, and their orientations and alignments, and the surgical approaches.^4^,^10^,^19^,^31^,^33^ Additionally, patient related factors such as gender, advanced age, history of previous hip surgery, pelvic tilt and bony structures around the hip increase the risk of impingement and subsequently recurrent dislocation.^8^,^10^,^11^,^33^ Bartz *et al.*^1^ classified dislocation mechanisms into three categories based on impingement type as follows: (a) prosthetic impingement (PI) which occurs when the prosthetic femoral neck comes in contact with the rim of the liner/cup, (b) bone-to-bone impingement (BTBI) which is the impingement between the osseous femur and the osseous pelvis, and (c) spontaneous dislocation. Although the factors which are associated with spontaneous dislocation are not fully identified, it is presumed that the soft tissue imbalance, weakness of the muscle, or/and contracture of the hip joint might increase its risk.^33^ Other than these three types, there is a possibility of implant-to-bone impingement (ITBI) which occurs when the prosthetic femoral stem comes in contact with pelvis or the bony femur comes into contact with the rim of the liner/cup. PI is associated with the acetabular and femoral implants only and has a known set of variables such as implant design and their positions and orientations on native bone geometries. Therefore, PI could be controlled with these known variables using a numerical simulation model to find optimal implant positions 23,27 or to select the optimal design (e.g., larger size of femoral head).^3^,^14^ BTBI, on the other hand, generally differs amongst subjects. It mainly depends on the bony structures around the hip along with the level of osteotomy, and the prosthetic geometries and their orientations (e.g., femoral offset and version *etc*.).^31^,^33^ It occurs due to unintended contact between femoral bones (e.g., greater trochanter, lesser trochanter, femoral neck) and acetabular margin (which is called “limbus acetabuli”), ilium or ischium (the anterior superior iliac spines is part of the ilium).^11^,^32^,^35^ ITBI could be partially controlled by changing implant designs and their positions and orientations. Therefore, it is usually recommended to recreate genuine bone morphology by resecting osteophytes completely during THA to avoid post-THA BTBI and ITBI.^14^,^21^,^31^,^33^ However, the risk assessment of post-THA BTBI and ITBI is mostly carried out intra-operatively by the surgeons based on their anatomical knowledge in recognising the difference between “genuine” and “osteophytic” bone. Therefore, it depends on the surgeons’ experiences, and intuitive anatomical reasoning, which are subjective and qualitative in nature. As a result, despite using recommended implant positioning along with resecting the bony prominence and osteophytes, post-THA complications related to impingement still occur for some cases, especially in patients with larger bony prominence.^33^ Pre-operative surgical planning is routine practice before THA, with some systems incorporating a computer simulation to predict optimal implant orientations and to explore post-THA hip joint ROM.^9^,^22^,^23^,^27^,^30^,^34^,^36^ However, the majority of the studies explored only the effect of implant design and position on PI.^23^,^27^,^34^,^36^ There are limited studies in the literature focusing on only BTBI,^9^,^12^,^25^,^33^ which mainly explored the effect of implant design and positions, bone morphology and hip joint ROM on BTBI. However, to the best of the authors’ knowledge, pre-operative identification of subject-specific bony impingement (BI) areas which should be resected to avoid post-THA BTBI and ITBI for a given implant design and position has not previously been reported in the literature.

Therefore, the aim of the paper was to develop a method for identifying subject-specific post-THA BTBI and ITBI areas, and subsequently visualise the impingement area on native bone anatomy. This novel visualisation representation could guide surgeons in deciding how much and from where the bony areas should be resected during THA to avoid bony impingement (BI) (i.e., both BTBI and ITBI) for particular implants and their given positions and orientations. The paper is structured as follows. A detailed description of the conceptual novelty followed by the implementation of the proposed method are included in the first part of materials and methods. Thereafter, a case study and a clinical study are included to describe the various features and validation of the method respectively. The rest of the paper describes the results followed by discussion.

## Materials and Methods

### Conceptual Novelty: Identification and Visualisation of the BI Area

**Figure 1:**
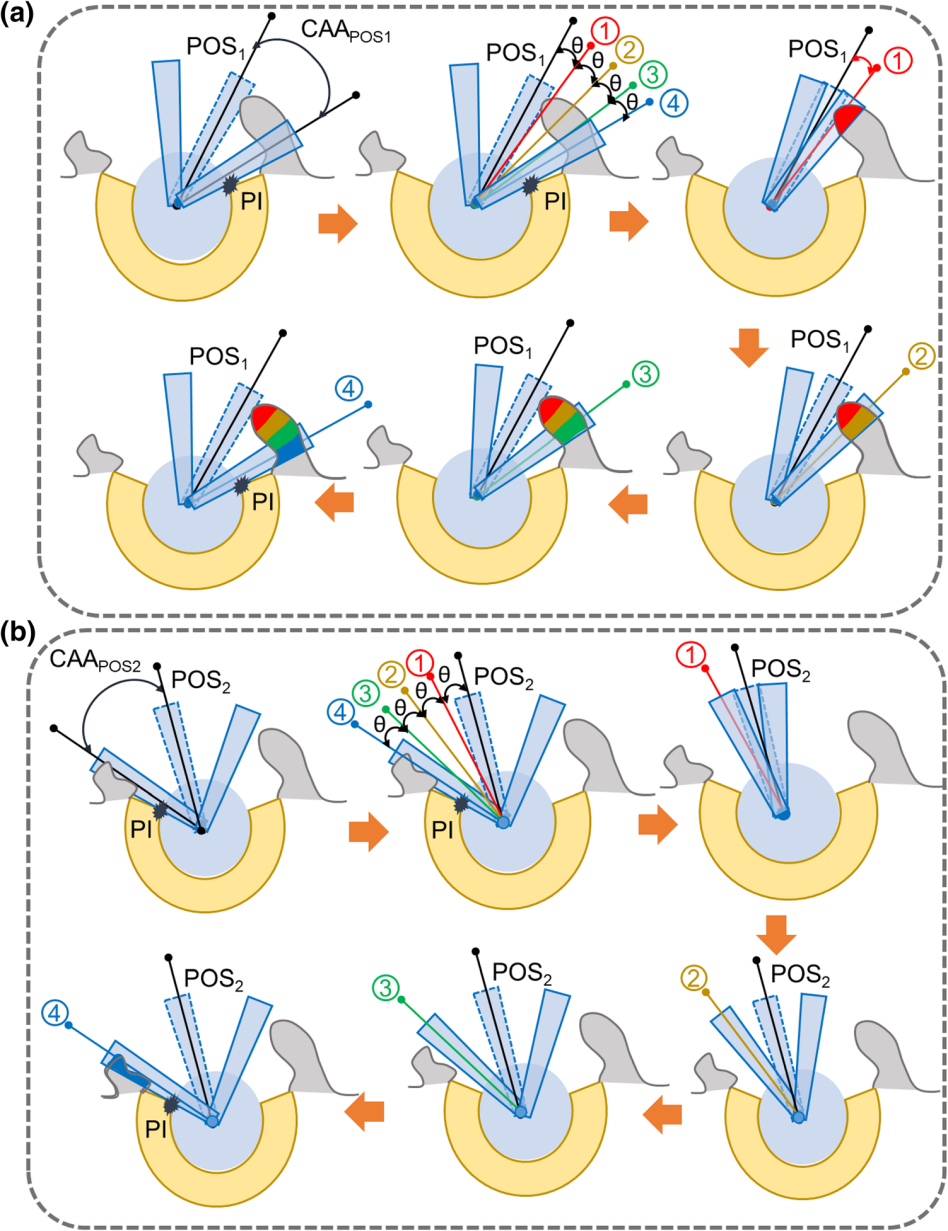
Schematic representation of the proposed concept for identifying and visualising bony impingement (BI) area. (a) POS_1_ and (b) POS_2_ represent two different postures. (a) ${\text{CAA}}_{{{\text{POS}}_{ 1} }}$ and (b) ${\text{CAA}}_{{{\text{POS}}_{ 2} }}$ are two ‘conical clearance angle’ associated with POS_1_ and POS_2_ respectively to avoid PI. 1, 2, 3, and 4 represent 25, 50, 75 and 100% of (a) ${\text{CAA}}_{{{\text{POS}}_{ 1} }}$ and (b) ${\text{CAA}}_{{{\text{POS}}_{ 2} }}$ respectively.

The proposed method is based on the following assumption that only the BTBI or ITBI which occurs before the PI is critical and these bony areas should be identified so that they could be resected during THA (Fig. 1). If these bony areas are resected, BTBI or ITBI can then only occur if the hip moves beyond the point of PI. However, this additional hip movement will be generally restricted by PI. Therefore, the first objective was to find the maximum clearance between stem and liner to avoid PI for a particular posture (Fig. 1). This clearance angle could be described as ‘Conical Clearance Angle’ (CCA) associated with that particular posture (Fig. 1). The next step was to find the bony impingement (BI) area which would occur within the ${\text{CAA}}_{{{\text{POS}}_{1} }}$ or ${\text{CAA}}_{{{\text{POS}}_{2} }}$ for posture POS_1_ and POS_2_ respectively. The chances of BI was ranked based on its occurrence within the range of ${\text{CAA}}_{{{\text{POS}}_{i} }}$ (*i *= 1, 2). The highest risk area was defined as any BI that would occur within the first 25% of ${\text{CAA}}_{{{\text{POS}}_{i} }}$ (position 1 in Fig. 1), and this area was highlighted with red (Fig 1a). Similarly, 50, 75 and 100% of ${\text{CAA}}_{{{\text{POS}}_{i} }}$ (*i* = 1, 2) were shown by positions 2, 3, and 4 respectively (Fig. 1). The corresponding BI areas were highlighted with different colours (yellow, green and blue respectively). Similar operations would be performed for different postures (Figs. 1a and 1b). It could be observed that for POS_1_, chances of BI were high as it occurred within 25% of ${\text{CAA}}_{{{\text{POS}}_{1} }}$ (Fig. 1a) whereas chances of BI for POS_2_ (Fig. 1b) was much less as it occurred in between 76 and 100% of ${\text{CAA}}_{{{\text{POS}}_{2} }}$.

### Implementation of the Method

#### Inputs

**Figure 2:**
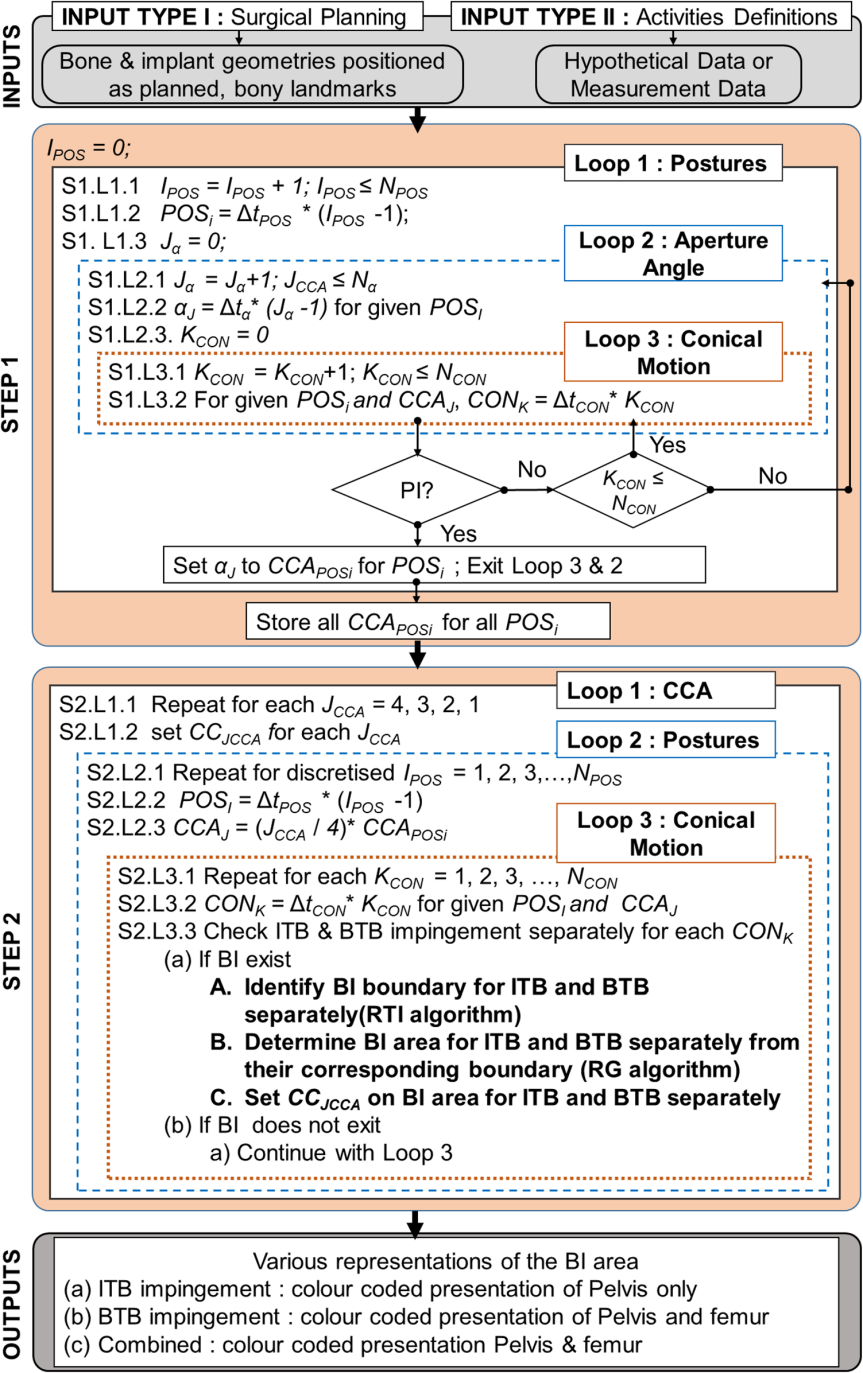
A brief overview of the proposed method to identify and visualise BTBI and ITBI. All variables in this flow diagram are defined in the main text.

The inputs, required for the proposed method, were broadly classified into two groups (Fig. 2). Input Type-I included both prosthetic implants with planned positions and native bone geometries. Therefore, Input Type-I was associated with the THA planning, and the following steps were performed to attain it: (a) CT scanning of a patient requiring THA surgery, (b) construction of subject-specific bone geometries from CT scans, (c) identification of bony landmarks by experienced engineers/surgeons, (d) CAD model of planned implants to be used for THA, and (e) planned implant positioning (e.g., cup/liner inclination and anteversion angle, stem offset *etc*.) onto the native bone structure. After all the aforementioned steps, the native bone geometries with planned implants, were used as Input Type I. In this work, STL file format with triangular mesh was used to represent both implants and bony structures. On the other hand, Input Type-II dealt with the hip joint motion under consideration. This hip motion could be either measured ADLs using gait analysis methods (i.e., through the use of video motion capture or inertial measurement sensors (IMUs), or generalised hypothetical activities from the literature such as pure/combined hip joint motion. Using these inputs, subject-specific BTBI and ITBI areas were identified as detailed below.

#### STEP 1: Determine Posture Specific Maximum Clearance to Avoid PI

**Figure 3:**
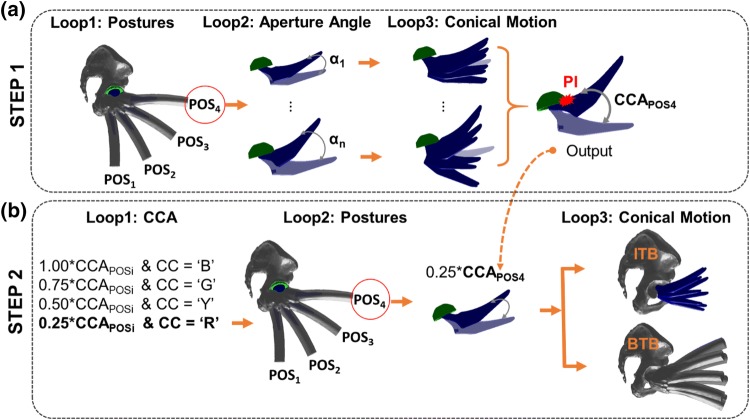
A graphical overview of different loops involved in STEP1 and STEP2, and the output of STEP 1 which is used in STEP2. CC, B, G, Y and R represent colour code, blue, green, yellow and red respectively.

The objective of this step was to determine the maximum amount of clearance between stem/neck and liner during a hip joint motion to avoid PI. This entire step was governed by three main loops as follows: (a) ‘Loop1: Postures’, (b) ‘Loop2: Aperture Angle’, and (c) ‘Loop 3: Conical Motion’ (Fig. 2-STEP1). ‘Loop1: Postures’ was associated with the hip joint motion under consideration which was discretised into a number of user defined postures (*N*_POS_) with a resolution of ∆*t*_POS_. For example, flexion up to 90° could be discretized with *N*_POS_ = 4 and ∆*t*_POS_ = 30° so that the femur flexion could be represented with four postures by varying counter *I*_POS_ such that POS_*i*_ = 0°, 30°, 60°, 90° for *I*_POS_= 1, 2, 3 and 4 respectively (Fig. 3a). For each posture, a hypothetical conical motion of femur was constructed where the axis of this conical motion was the femur axis at this particular posture. Within the same posture, and therefore, using the same conical axis, various conical motions were created by varying its aperture angle (*α*) to check the degree of clearance between stem/neck and the liner to avoid PI. The limiting/maximum aperture angle (*α*) of the conical motion, which would avoid PI, was hypothesized as a ‘Conical Clearance Angle’ (CCA). The increment in *α* to find CCA was governed by ‘Loop2: Aperture Angle’ where *N*_*α*_ was the user-defined number of discretised *α* with a resolution of ∆*t*_*α*_. Therefore, varying *α*_J_ was calculated as Δ*t*_*α*_ *(*J*_*α*_ − 1) where *J*_*α*_ was the counter of the loop. For example, if Δ*t*_*α*_= 1°, *α*_J_ value would increase by 1° with increase of counter *J*_*α*_. Similarly, if Δ*t*_*α*_= 5°, *α*_J_ value would increase by 5° with single increment of counter *J*_*α*_. The conical motion of femur with a particular *α*_*J*_ was discretized with *N*_CON_ number of positions with a resolution of *Δt*_CON_ where CON_*k*_ (CON_*K*_ = *Δt*_CON_ * *K*_CON_) represented each position of femur during this conical motion and *K*_CON_ was a counter of the loop (Fig. 2). Figure 3a shows that the conical motion is discretized with *N*_CON_ = 8 static positions. For each of the CON_*k*_ position, PI was checked. If there was PI, the associated *α*_J_ value was considered as the ${\text{CAA}}_{{{\text{POS}}_{i} }}$ for that particular posture (POS_i_). Subsequently, the execution of Loop3 and Loop2 were terminated and Loop1 started with each next posture (POS_*i*_). If there was no PI occurring even after completing Loop3 and then Loop2, the ${\text{CAA}}_{{{\text{POS}}_{i} }}$ for that posture was considered as the user-defined maximum *α*_J_ value, that is Δ*t*_*α*_ * (*N*_*α*_ − *1*). PI was checked by using Matlab function ‘fastMesh2Mesh’ 29 which was developed based on Möller-Trumbore’s (MT) ‘ray triangle intersection’ (RTI) algorithm.^17^

Therefore, STEP 1 ended when Loop 1 ended and posture specific ${\text{CAA}}_{{{\text{POS}}_{i} }}$ for all the postures were determined.

#### STEP 2: Determine BI Areas

**Figure 4:**
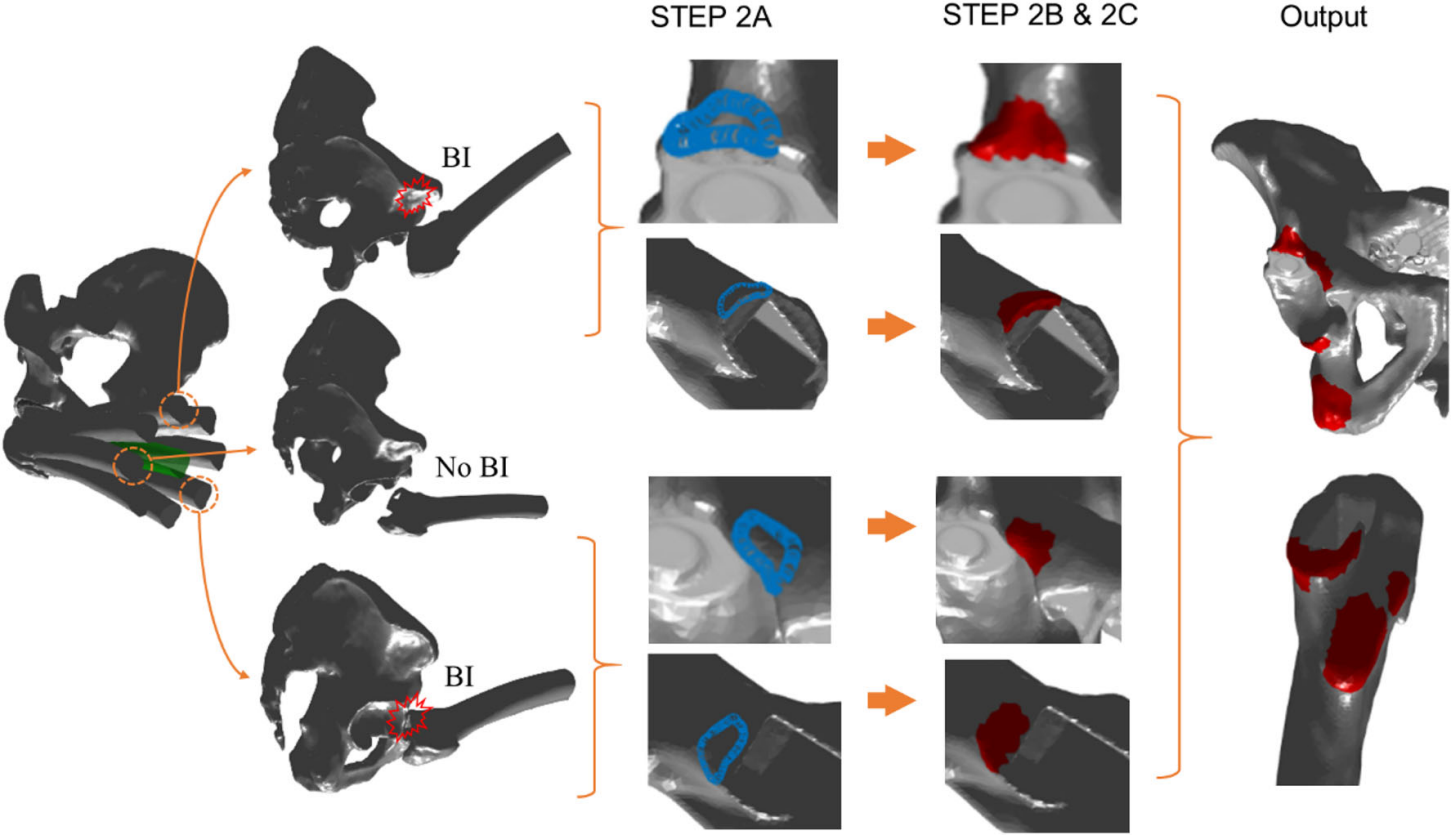
A brief overview of STEP 2A, B and C along with final output.

The objective of the step was to identify and determine the BI area which would occur within the ${\text{CAA}}_{{{\text{POS}}_{i} }}$, determined from the last step. For visualisation purposes, each ${\text{CAA}}_{{{\text{POS}}_{i} }}$ was divided into four categories to rank the potential chances of BI as follows: (I) 100%, (II) 75%, (III) 50% and (IV) 25% of ${\text{CAA}}_{{{\text{POS}}_{i} }}$ where 100 and 25% represented the lowest and the highest risk area respectively (Fig. 1). For each posture, BI was checked for each category of ${\text{CAA}}_{{{\text{POS}}_{i} }}$. Therefore, the highest risk area would be associated with the BI area if it occurred within the 25% of ${\text{CAA}}_{{{\text{POS}}_{i} }}$. Similarly, the risk would be gradually reduced if BI occurred within 50, 75 and 100% of ${\text{CAA}}_{{{\text{POS}}_{i} }}$. In terms of visual representation, BI areas were presented with user-specific colours. In this work, red, yellow, green and blue depicted 25, 50, 75 and 100% of ${\text{CAA}}_{{{\text{POS}}_{i} }}$, and thus representing from higher to lower chance of BI occurrence (Fig. 1). This step was also governed by three loops (Figs. 2 and 3b). In ‘Loop1: CCA’, CC_JCCA_ stored the colour code (i.e., red, yellow, green and blue) for each CCA category. ‘Loop2: Posture’ executed different postures that were obtained by discretised hip joint motion under consideration, which was exactly the same posture generated in STEP1- ‘Loop1: Postures’. For each posture, posture specific CCAs categories were obtained for (CCA_J_= (*J*_CCA_/4) * ${\text{CAA}}_{{{\text{POS}}_{i} }}$). ‘Loop3: Conical Motion’ performed the conical movement similar to the STEP1. For each CCA category, each posture, and each conical motion position, BI was checked and identified (if any), and depicted using three following sub-steps (Fig. 4).

#### STEP 2A: Check and Identify BTBI and ITBI Boundary for Particular CCAs

For each position of the conical motion, intersection between two geometrical structures was calculated (Fig. 4). Femur and pelvis geometries were used for BTBI whereas stem/neck and pelvis geometries were used for ITBI. Möller–Trumbore (MT) ‘ray triangle intersection’ (RTI) algorithm 17 was used to find the intersection boundary points between two STL geometries with triangular mesh. In this work, the Matlab function ‘fastMesh2Mesh’, developed by Thomas Seers based on Möller–Trumbore algorithm, was used for calculating the intersections boundary points.^29^ The output (intersection points) from this Matlab function were compared with the intersection points, calculated by Mimics 3-matic software when used same STL geometries. It was observed that both the output are similar, and therefore, the Matlab function was used thereafter for the ease of automation.

#### STEP 2B: Determine BTBI and ITBI Area

**Figure 5:**
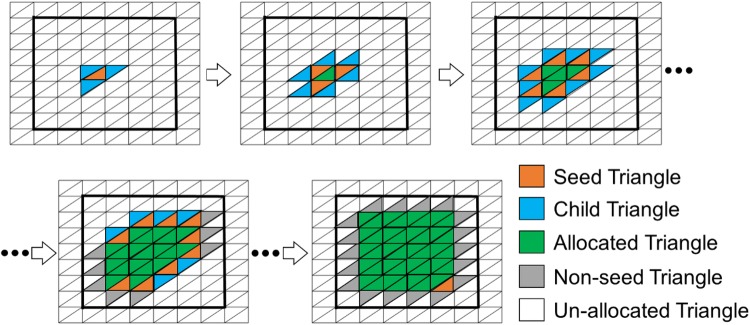
A schematic representation of the region growing algorithm (RGA).^6^

This step included a region growing algorithm (RGA) which was originally developed by Franciosa and Gerbino 6 for planar and cylindrical features recognition in a CAD-based application. This RGA was further used in ventricular geometry to assign fibre orienation.^20^,^24^ In this work, RGA was used to automatically calculate the surface area confined within this BI boundary, identified in the last step. The RGA was carried out by proliferating the ‘child’ triangles around the ‘seed’ ones. A triangle was defined as a ‘seed’ triangle if all three of its vertices belonged to the same cluster (Fig. 5). On the other hand, a triangle was termed ‘child’ if it followed two conditions: (a) the triangle was itself a ‘seed’ triangle, and (b) it had a common (sharing) edge with a neighbouring ‘seed’ triangle of the same cluster (Fig. 5). Figure 5 represents a typical propagation mechanism associated with RGA where the squared area, enclosed by thick black lines, could be thought as a BI boundary. The RGA started with a random triangle which was considered as the first ‘seed’ triangle and associated with the first cluster. The ‘child’ triangles around a ‘seed’ triangle were then identified, and thus, the region growing started. In the next step, the identified ‘child’ triangles were considered as ‘seed’ triangles, and old ‘seed’ triangles (‘seed triangles in the previous step) were defined as ‘allocated’ triangles within the same cluster (Fig. 5). Subsequently, new ‘child’ triangles were identified around the new ‘seed’ triangles, and this process continued until there was no ‘child’ triangle (un-allocated triangles) available to be assigned within the same cluster. As a result, the region was grown around a ‘seed’ triangle confined within a closed boundary, and stopped automatically near to the edge of the boundary (in this case, BI boundary) (Fig. 5). The vertices of triangles at boundary edges were shared between two different clusters (regions), and therefore, these triangles were not considered as ‘seed’ as all three vertices were not within a same cluster/region (Fig. 5). Therefore, the region identified using RGA within BI boundary would be a little smaller than the actual area (Fig. 5). However, the differences would be negligible if small triangular mesh is used to construct the bony geometries. The entire RGA stopped when there was no ‘seed’ triangle available in the entire geometry, i.e., there was no new cluster to be created. After applying RGA, the entire geometry (femur or pelvis) was clustered into two areas: (a) impinged areas, and (b) non-impinged areas. The impinged triangular face ids (*ITFs*) were recorded, and stored for future use. The same operation was carried out separately for BTBI and ITBI analysis.

#### STEP 2C: Assign Colours to BTBI and ITBI Area

At the beginning of the STEP 2, all the triangular faces of the femur and pelvis were assigned with a user defined ‘non-impinged colour’ (e.g., grey), that differed from the four colours used for BI area representation. After STEP 2B, BI area was identified, and ITFs were recorded (Fig. 4). A user defined colour (CC_JCCA_) was then assigned to these ITFs whereas the rest of the triangular faces remained with ‘non-impinged colour’ (grey) (Fig. 4). This user defined colour (*CC*_*JCCA*_) was associated with the CCA classification, which was categorised to rank the chances of BI. This process was carried out separately for BTBI and ITBI analysis. For ITBI analysis, the entire stem geometry was used to check for impingement. However impingement is only expected around the neck of the stem. This is due to a large percentage of the stem being located within the femoral canal, i.e., covered by the bone and hence would not come into contact with the pelvis. Therefore, some of the ITFs, which was predicted due to the collision of this region of stem to pelvis, would be infeasible. These ITFs were identified by checking the common ITFs from both BTBI and ITBI analysis. The colour of these common ITFs were changed to ‘a ‘non-impinged colour’ (grey) only for ITBI representation.

#### Outputs

After STEP 2, BI areas of the pelvis and femur were identified through ITFs with a CCA specific colour. These BI areas were represented in three styles. The ITBI information was presented only by showing the colour map on the pelvis, whereas BTBI areas were shown both on the pelvis and the femur. The last option was to combine both ITBI and BTBI together.

### Case Study

The surgical plans for a right hip joint was used to demonstrate the various features of the proposed method. Input Type I for the method (Fig. 2) was provided by experienced engineers at Corin Ltd, who provide a THR planning service for orthopaedic surgeons. This retrospective analysis of the data from Corin was approved by Bellberry Human Research Ethics Committee (BHREC)—study number 2012-03-710 and Biomedical & Scientific Research Ethics Committee (BSREC) in Warwick University (study reference REGO-2018-2229). The acetabular components positions were defined by radiographic inclination and anteversion angles, as defined by Murray,^18^ which were within the safe zone.^13^ For Input Type II, four hypothetical activities 23 were used (Table 1). These activities were typically performed during surgery to check for potential impingement.

**Table 1:** A summary of activity definitions (Input Type II) used for the study.^23^

Activities	Initial position	Final position
Extension (Extn)	Supine	10° Extn
Flexion (Flex)	Supine	90° Flex
External rotation at extension (ER_Ext_)	10° Extn, 0° ER_Ext_	25° ER_Ext_
Internal rotation at flexion (IR_Flex_)	90° Flex, 0° IR_Flex_	35° IR_Flex_

### Clinical Study and Validation of the Method

In order to validate the method, the data of the patients, who had previously undergone a THA without the help of the proposed method, was used in this study. The anonymised data, provided by Corin Ltd, was approved by BHREC (2012-03-710) and the retrospective study was approved by BSREC (REGO-2018-2229). Five anonymised patients were selected based on the criteria that there had been no postoperative episodes of hip dislocation, with a follow up of at least 2 years. It was therefore assumed that the cases did not experience PI or BI post-operatively and hence, the bony areas, which were resected intraoperatively could be used as a benchmark for comparison. Table 2 summarises the patient characteristics and related intraoperative data.

**Table 2:** Patient characteristics and intraoperative data.

Characteristic	Patient (*n* = 5)
Sex (male/female)	3/2
Age (years)	68.10 ± 10.20
Treatment Side (right/left)	2/3
Cup size (diameter in mm)	53.20 ± 2.28; (50–56)
Head size (diameter in mm)	32.00 ± 0.00
Cup inclination (°)	38.40 ± 5.17; (32–46)
Cup anteversion (°)	16.40 ± 3.97; (11–22)
Stem anteversion (°)	13.80 ± 11.52; (0.01–25.38)

3D models of the used implants and their positions within the native bone geometries, as implemented during surgery, were used as Input Type I. This information was extracted from the post-operative CT scan by dedicated experienced engineers in Corin Ltd. For Input Type II, four hypothetical activities were selected (Table 1). Both pre-operative (Pre-Op) and post-operative (Post-Op) geometries of the pelvis and femur were used for the prediction of both the ITBI and BTBI area. As some of the areas in the Post-Op geometry was already resected during surgery, it would be expected that the ITBI and BTBI area, predicted using Pre-Op geometries would be much higher than the ITBI and BTBI area identified from Post-Op geometries. For numerical representation, the reductions in coloured surface areas (red, yellow, green and blue) from Pre-Op to Post-Op geometry were calculated for ITBI and BTBI cases. The higher the reduction percentage (best is 100% reduction), the better the prediction from the method. Therefore, the study hypothesis was—‘At least the red impingement area (i.e., 25% of ${\text{CAA}}_{{{\text{POS}}_{i} }}$ which was the most critical area) should have been resected, and therefore, this area (entirely or partially) should not exist in Post-Op geometries’.

## Results

### Case Study

**Figure 6:**
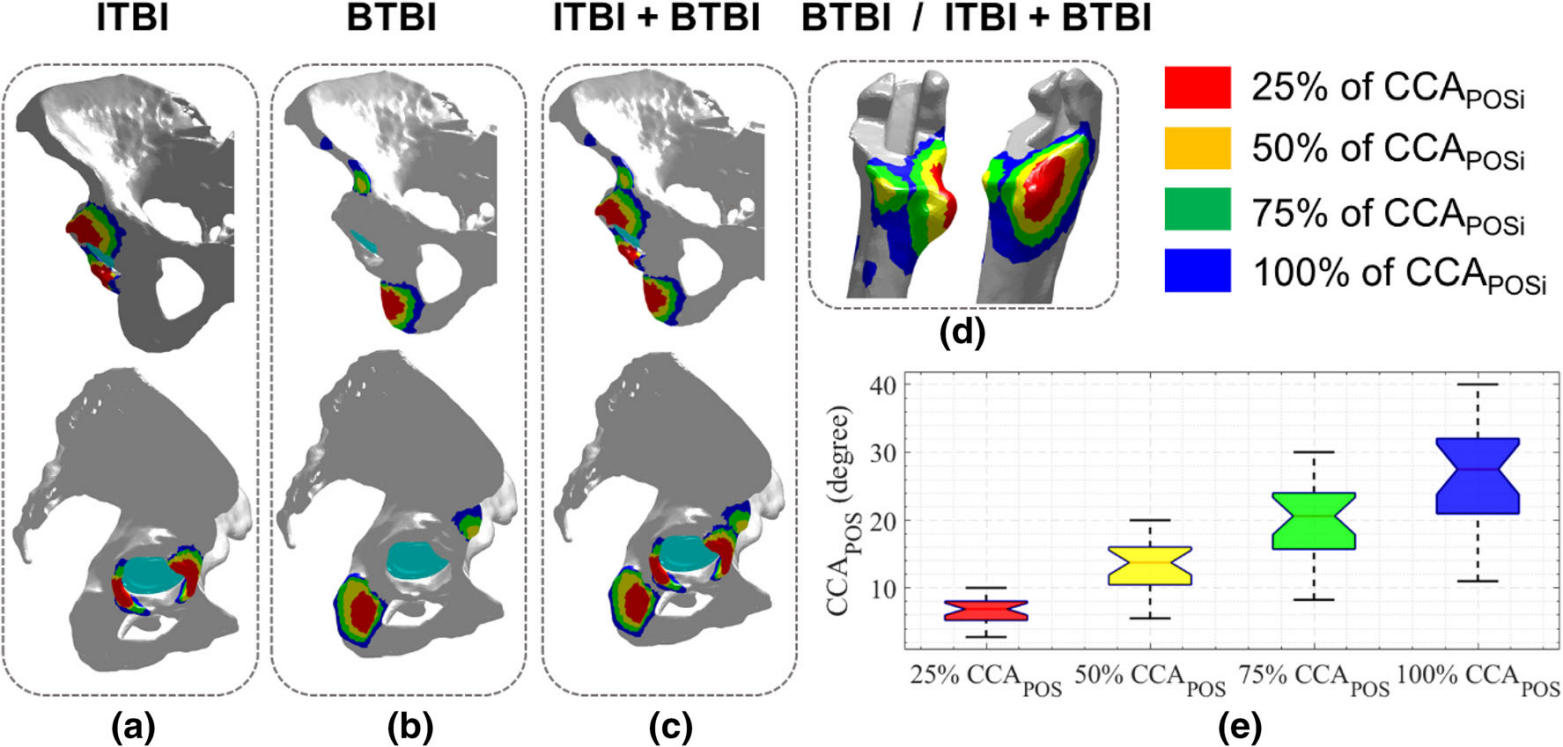
Representation style of ITBI and BTBI information through colour coded area.

There were three representation type—(a) ITBI where only pelvis geometry was used (Fig. 6a), (b) BTBI where both pelvis and femur geometries were used (Figs. 6b and 6d), and (c) combined, i.e., both ITBI and BTBI presented together on pelvis and femur (Figs. 6c and 6d). From the results, it was observed that the ITBI area was near to the acetabulum that was generally resected during surgery. On the other hand, the location of the predicted BTBI area was not quite common from where the resection was generally done. The combined plot included both ITBI and BTBI information, and could be used in surgical planning without getting into details of type of BI. It should be noted that the bony areas could be resected either from the pelvis or femur to avoid BTBI, although surgeons generally prefer to resect osteophyte bone from pelvis.

The red areas (Fig. 6) depicted the highest risk area as this would occur only within 25% of the PI clearance (${\text{CAA}}_{{{\text{POS}}_{i} }}$). This area should be resected during surgery if these planned implant positions (cup positions and stem offset *etc*.) would be actually realised. On the other hand, the minimum risk area was depicted by blue areas where the BI would occur beyond 75% and above PI clearance. The yellow and green zone highlighted the BI area due to 26–50% and 51–75% of PI clearance respectively. This coloured representation could be easily comprehended by the surgeons before making their decision of bony resection. Figure 6e represented the distribution of CCA associated with the various postures. This was the output from STEP1 of the method.

### Clinical Study and Validation of the Method

**Figure 7:**
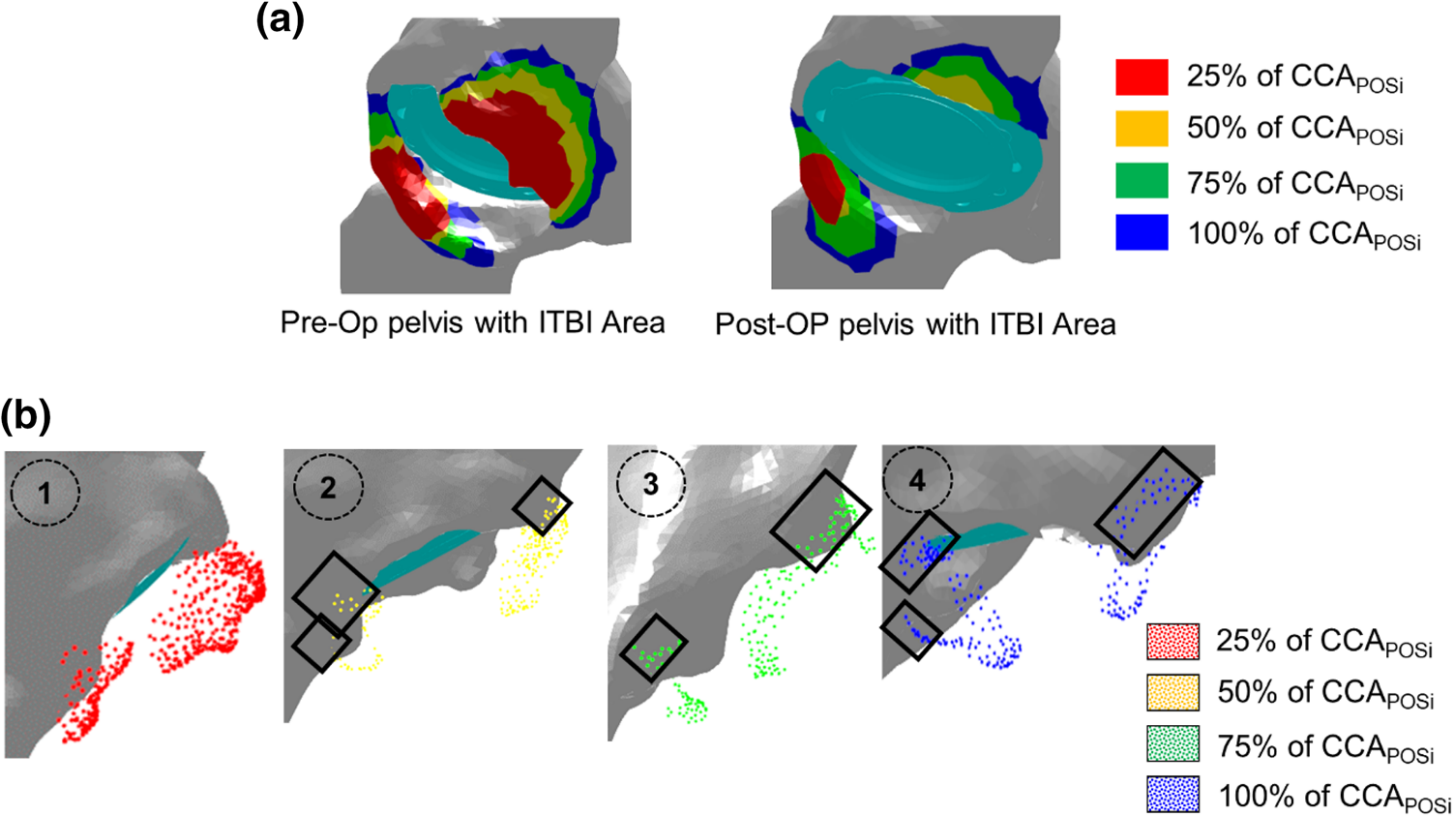
A brief overview of the validation method. (a) The ITBI area was identified using both Per-Op and Post-Op geometries; (b) Comparison through visual representation of bony areas which were resected during surgery (Post-Op) compared to the predicted ITBI area using Pre-Op geometry. The grey geometry is Post-Op pelvis. The coloured points are the point cloud of the ITBI area predicted through the method using Pre-Op geometry. The square box shows the points on the pelvis, i.e., these points were not resected during surgery.

The ITBI area was calculated using both Pre-Op and Post-Op pelvis (Fig. 7a). It was observed that the red and yellow area was reduced considerably in the Post-Op pelvis compared to Pre-Op geometry, which suggested that those area were mainly resected by the surgeons during surgery. A more detailed observation was made through Fig. 7b where the location of the coloured points, which represented the predicted ITBI area using Pre-Op pelvis (Fig. 7a), were compared with the Post-Op pelvis. It was observed that the majority of the red and the yellow points were located outside of the pelvis geometry. This confirmed that these areas were resected during surgery. On the other hand, the green and the blue points were partially located on the pelvis, and therefore, these areas were partially resected. The coloured points within the black boxes (Fig. 7b) were still located on pelvis. It showed that these areas were not resected.

**Figure 8:**
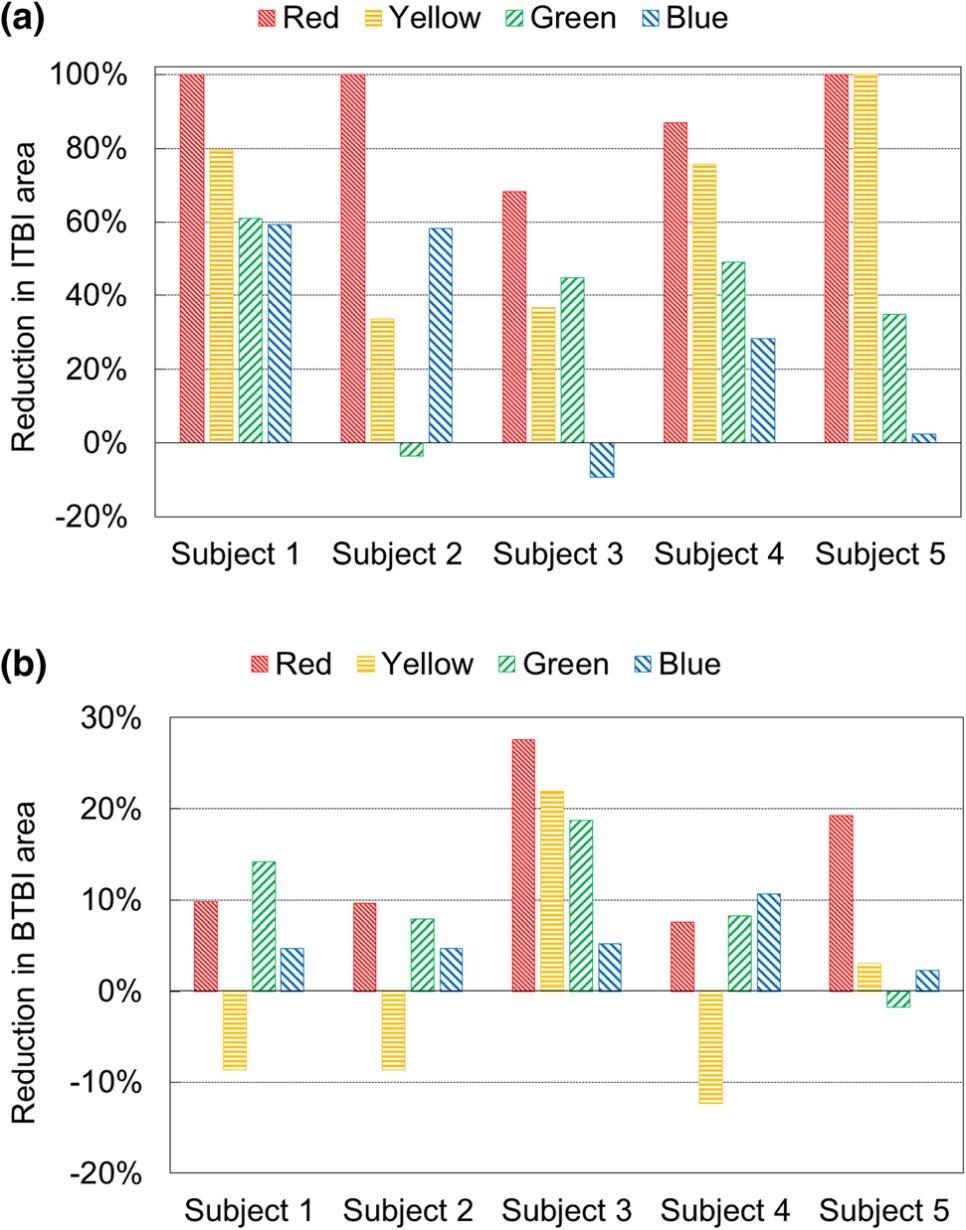
Reduction in coloured (a) ITBI and (b) BTBI areas from Pre-Op to Post-Op geometry to compare the actual amount of resected bony area with respect to predicted area.

The reduction in surface area from Pre-Op to Post-Op geometry for each coloured zone was calculated (Fig. 8) to compare the prediction of the proposed method with respect to the actual resection done intraoperatively by the surgeons. In case of ITBI (Fig. 8a), it was observed that the red area (highest risk zone) for three subjects were 100% removed, and for other two subjects, major areas were (87% and 68%) resected. This agreed well with the study hypothesis. Furthermore, yellow areas for one subject were 100% removed, whereas major areas were (79% and 75%) resected for two subjects. However, for the remaining subjects, the reduction was not considerable (only 33.6% and 36%). The reductions in green and blue areas, where the chances of impingement were very low, were less than 50% for all subjects except for subject 1 (for both green and blue) and subject 2 (blue only). For the case of BTBI, there was no strong evidence of reduction of any of the coloured areas from Pre-Op to Post-Op geometry as the reduction percentages were quite low (less than 30%) (Fig. 8b). The minor negative reductions (Fig. 8) depicted that there were additional areas which were not present in the Pre-Op geometry. This could be due to segmentation and alignment errors, as discussed in detail in discussion section.

## Discussion

BI is one of the major causes of a constrained hip joint motion and recurrent dislocation after THA. The current practise of BI risk assessment is performed intraoperatively by surgeons based on their expertise knowledge and experience, which sometimes lead to suboptimal surgical outcome. This paper introduced a novel method to identify and visualise both ITBI and BTBI area on native bone geometry for different hip joint motion under consideration. In addition, the chances of BI was ranked, and represented with different coloured areas on pelvis and femur. This coloured representation of BI areas could be used as a guideline to pre-plan the surgery in order to decide how much and from where the bony resection should be performed to avoid post-THA BI for a particular implants and their given positions. The proposed method has various features that might be modified according to the user’s requirement as follows. Firstly, the possibility of BI was grouped into four categories, which was further represented by four different colours. However, this was entirely user specific. The user could use any colour and any number of categorisations based on their requirements. Besides, it could also be possible to inform which activity causes red-coloured BI site through different shade of red colours or simply by using a list of such activities. Secondly, the box plot of CCA provided the information about PI. If there is a PI for zero CCA, it means that there is a PI due to the hip joint motion itself. Therefore, BI representation with a box-plot of CCA would provide both BI and PI information for a subject with given implant positions. Thirdly, the BI area could be represented either by ITBI or BTBI (for detailed analysis) or combining both (for easier interpretation). Any bony resection either from the pelvis or the femur would help to avoid BI. Although it is not common to perform resection from femur, the surgeons would get additional information which they might use in future where resection from the pelvis wouldn’t be feasible or complicated. Fourthly, bony resection may not always be desirable or feasible, and therefore, corrective strategies may need to be considered to reduce the BI area, such as using different implant combinations, positions and offset *etc*. The proposed method in the paper could be used to re-evaluate the effect of any such implant changes on the BI area. The corrective actions such as change in leg length or medio-lateral offset or the type of implants are the part of surgical planning, and therefore, any such corrective actions would be conveyed through the implant geometries and positions which are nothing but Input Type I of the method. Therefore, the surgeons can plan for various (three or four or more) sets of corrective actions and run the simulation for each set of corrective actions. Subsequently, the results could be compared in terms of bony resection needs to be performed. If any of the corrective action shows that the amount of bony resection is less and also feasible, the surgeons could finalise the corrective actions provided this would not compromise with other biomechanical issues such as stability of the joint or edge loading *etc*. which this method does not consider. Fifthly, the resolutions of discretizing activity (*Δt*_POS_), aperture angle (*Δt*_*α*_) and conical motion (*Δt*_CON_) were important factors. Higher resolution would provide accurate results with higher computation expenses. However, there would be some redundant postures or positions for higher resolution that would not alter the final BI area. On the other hand, increasing the value of *Δt*_POS_ and *Δt*_CON_ could underestimate the BI area. Therefore, a trade-off value should be elected so that an accurate BI area could be identified with reasonable computational time. For the case study in the paper, i.e., four hip motions with *Δt*_POS_ = 5°, *Δt*α = 1°, and *Δt*_CON_ = 10°, it generally took 5 to 6 h to generate the interference map from Input Type I and II in a computer with 64 GB RAM, and 8 core Intel Xeon E3-1535 M v6 (3.10 GHz) processor. CT scanning and preparation of Input Type I required approximately 1 to 2 h of additional time. Therefore, it would take generally 6 to 8 h from CT scan to colour map generation. However, the entire process is still not optimised, and therefore, there is a potential to reduce the overall time below 5 h. Most importantly, as these would be generally carried out in planning stage of the THA, 6 to 8 h would still not be very critical, and would not cause any unnecessary delays in surgery. Sixthly, although the proposed method was originally developed for checking post-operative BTBI and ITBI for a given implant position and orientation, this technique could potentially also be used by surgeons addressing femero-acetabular impingement (FAI). However, in that case, no implant geometries and their positions are required. Finally, the values of the hip joint motions or the implant orientations used in this work were typical demonstrative values within their ranges as recognised from the literature. However, the proposed method is not restricted to these values only.

The validation was performed by checking the reduction percentage of coloured surface area from Pre-Op to Post-Op geometries. However, it should be noted that the reduction percentage not only included actual bony resection, but also involved alignment and segmentation errors. The Pre-OP and Post-Op CT scans were performed with at least 2 years gap and the developed geometries were in different coordinates. However, all the implant positions were defined with respect to Pre-Op geometry. Therefore, a best-fit alignment was performed (moving Post-Op geometry to Pre-Op location) so that the BI analysis could be carried out on Post-Op geometry using the implant positions which were originally defined with respect to Per-Op geometry. This best fit alignment introduced additional error in identifying BI area using Post-Op geometry. Besides, during Post-OP CT scans, the radiation bounced off the implants, and the resulting artefacts decreased the spatial resolution of the scanned bone geometry around the implant. As a result, the segmentation from the Post-Op CT scan was difficult due to the indistinct boundary of bone geometry. Also, various filters and smoothing algorithms were applied by radiology department prior to segmentation, which also led to differences in Pre-OP and Post-OP CT scans. As a result, the developed Pre-Op and Post-Op geometries were not exactly same where resection was not performed. For these reasons, reduction percentage in coloured surface area from Per-Op to Post-Op would be the combined results of (a) actual resection, (b) alignment error, (c) segmentation error, and (d) differences in CT scans. Therefore, the reduction percentage, which theoretically should only be due to surgical resection, might be over or under estimated. These scenarios explicitly observed when there was some negative reduction for both ITBI and BTBI analysis (Fig. 8). For this reason, a qualitative-numerical validation was carried out. Each reduction was checked visually to confirm whether the reduction is due to the actual bony resection. From the results, it was observed that the reduction in red area was near to 100%, and when checked visually, there was a little red area on Post-Op geometry. It was concluded the reduction was mostly due to resection. However, for the same reason, no conclusion could be drawn from green and blue areas of ITBI analysis and entire BTBI analysis. Specifically, the reduction percentage was very low for BTBI analysis and there was no strong visual confirmation as well. Therefore, it was undecided whether the 20 or 30% was due to the actual resection or some additional error mentioned above. The red areas due to BTBI were not resected as these areas were not so common for resection during surgery. However, the predicted ITBI and BITBI areas were due to same CAA. Therefore, resecting only ITBI area wouldn’t avoid the BI entirely. If the hip joint motion reached to the range of CCA, there might not be any ITBI but there would be BTBI. Therefore, these uncommon BTBI areas should also be removed to avoid BI completely.

One of the limitation of the study was to use four hypothetical activities. These were defined based on the generalised ROM data from the literature due to unavailability of subject-specific data. However, the ROM, which could be produced due to other activities, were partially covered by the conical motion as it created additional ROM for hip joint. Secondly, there was no direct validation of the method. It is not possible at present to accurately and directly record femoral movements or the presence of BI simultaneously in real-time in patients. Our indirect validation, performed through the clinical study, produced results which are consistent with clinical experience.

This paper presents a novel method to identify and visualise subject-specific ITBI and BTBI area on native bone geometries (femur and pelvis) for various hip joint motion under consideration. The method checks for a conical clearance for a set of postures during an activity to avoid PI, and subsequently, identity and visualise the BI area which would occur before PI. In addition, the BI area was ranked according to the chance of occurrence, and represented with different colour for improved understanding. This method could potentially be used to examine the effect of different pre-operative plans and hip motion on BI and partially on PI. In addition, this method would guide the surgeons to decide how much and where the bony resection should be performed during a THA surgery.

## References

[1] Bartz RL, Nobel PC, Kadakia NR, Tullos HS (2000). The effect of femoral component head size on posterior dislocation of the artificial hip joint. J Bone Joint Surg Am.

[2] Bennett D, Humphreys L, O’Brien S, Orr J, Beverland DE (2009). Temporospatial parameters of hip replacement patients ten years post-operatively. International orthopaedics.

[3] Crowninshield RD, Maloney WJ, Wentz DH, Humphrey SM, Blanchard CR (2004). Biomechanics of large femoral heads: what they do and don’t do. Clin Orthop Relat Res.

[4] D’Lima DD, Urquhart AG, Buehler KO, Walker RH, Colwell CW (2000). The effect of the orientation of the acetabular and femoral components on the range of motion of the hip at different head-neck ratios. J Bone Joint Surg Am.

[5] Enocson A, Hedbeck CJ, Tidermark J, Pettersson H, Ponzer S, Lapidus LJ (2009). Dislocation of total hip replacement in patients with fractures of the femoral neck. Acta Orthop.

[6] Franciosa P. and S. Gerbino. A cad-based methodology for planar and cylindrical features recognition. In: *4th CIRP International Conference on Intelligent computation in manufacturing engineering*2008.

[7] Huo MH, Parvizi J, Bal BS, Mont MA (2009). What’s new in total hip arthroplasty. J Bone Joint Surg Am.

[8] Jolles BM, Zangger P, Leyvraz PF (2002). Factors presdisposing to dislocation after primary total hip prosthesis. J Arthroplasty.

[9] Kessler O, Patil S, Wirth S, Mayr E, Colwell CW, D’Lima DD (2008). Bony impingement affects range of motion after total hip arthroplasty: A subject-specific approach. J Orthop Res.

[10] Kotwal RS, Ganapathi M, John A, Maheson M (2009). Jones SA Outcome of treatment for dislocation after primary total hip replacement. The Journal of Bone and Joint Surgery British.

[11] Lachiewicz PF, Soileau ES (2013). Low early and late dislocation rates with 36- and 40-mm heads in patients at high risk for dislocation. Clin Orthop Relat Res.

[12] Lerch TD, Degonda C, Schmaranzer F, Todorski I, Cullmann-Bastian J, Zheng G, Siebenrock KA, Tannast M (2019). Patient-Specific 3-D Magnetic Resonance Imaging-Based Dynamic Simulation of Hip Impingement and Range of Motion Can Replace 3-D Computed Tomography-Based Simulation for Patients With Femoroacetabular Impingement: Implications for Planning Open Hip Preservation Surgery and Hip Arthroscopy. The American Journal of Sports Medicine.

[13] Lewinnek GE, Lewis JL, Tarr R, Compere CL, Zimmerman JR (1978). Dislocations after total hip-replacement arthroplasties. J Bone Joint Surg Am.

[14] Malik A, Maheshwari A, Dorr LD (2007). Impingement with total hip replacement. J Bone Joint Surg Am.

[15] Malkani AL, Ong KL, Lau E, Kurtz SM, Justice BJ, Manley MT (2010). Early- and Late-Term Dislocation Risk After Primary Hip Arthroplasty in the Medicare Population. J Arthroplasty.

[16] Miki H, Sugano N, Yonenobu K, Tsuda K, Hattori M, Suzuki N (2013). Detecting cause of dislocation after total hip arthroplasty by patient-specific four-dimensional motion analysis. Clin Biomech (Bristol, Avon).

[17] Möller T, Trumbore B (1997). Fast, Minimum Storage Ray-Triangle Intersection. Journal of Graphics Tools.

[18] Murray DW (1993). The definition and measurement of acetabular orientation. J Bone Joint Surg Br.

[19] Nadzadi ME, Pedersen DR, Yack HJ, Callaghan JJ, Brown TD (2003). Kinematics, kinetics, and finite element analysis of commonplace maneuvers at risk for total hip dislocation. J Biomech.

[20] Palit A (2015). Computational modelling of diastole for human ventricle.

[21] Palit A., R. King, Y. Gu, J. Pierrepont, Z. Hart, M. T. Elliott and M. A. Williams. Prediction and Visualisation of Bony Impingement for Subject Specific Total Hip Arthroplasty*. In: *2019 41st Annual International Conference of the IEEE Engineering in Medicine and Biology Society (EMBC)*2019, p. 2127-2131. 10.1109/EMBC.2019.8857861 31946321

[22] Palit A, King R, Gu Y, Pierrepont J, Hart Z, Elliott MT, Williams MA (2019). Why is this hip replacement dislocating? A novel biomechanical prediction with 2d representation of edge loading and prosthetic impingement risk. Orthopaedic Proceedings.

[23] Palit A, King R, Gu Y, Pierrepont J, Simpson D, Williams MA (2019). Subject-Specific Surgical Planning for Hip Replacement: A Novel 2D Graphical Representation of 3D Hip Motion and Prosthetic Impingement Information. Annals of Biomedical Engineering.

[24] Palit A, Turley GA, Bhudia SK, Wellings R, Williams MA, Goh J (2014). Assigning Myocardial Fibre Orientation to a Computational Biventricular Human Heart Model. *The 15th International Conference on Biomedical Engineering*.

[25] Palit A, Williams MA, Turley GA, Renkawitz T, Weber M (2017). Femur First navigation can reduce impingement severity compared to traditional free hand total hip arthroplasty. Scientific Reports.

[26] Rowan FE, Benjamin B, Pietrak JR, Haddad FS (2018). Prevention of Dislocation After Total Hip Arthroplasty. J Arthroplasty.

[27] Schmid J, Chênes C, Chagué S, Hoffmeyer P, Christofilopoulos P, Bernardoni M, Charbonnier C (2015). MyHip: supporting planning and surgical guidance for a better total hip arthroplasty: A pilot study. International journal of computer assisted radiology and surgery.

[28] Scifert CF, Noble PC, Brown TD, Bartz RL, Kadakia N, Sugano N, Johnston RC, Pedersen DR, Callaghan JJ (2001). Experimental and computational simulation of total hip arthroplasty dislocation. Orthop Clin North Am.

[29] Seers T. Fast mesh-mesh intersection using ray-tri intersection with octree spatial partitioning. MathWorks File Exchange, 2015.

[30] Shoji T, Yamasaki T, Izumi S, Hachisuka S, Ochi MJIO (2016). The influence of stem offset and neck shaft angles on the range of motion in total hip arthroplasty..

[31] Shoji T, Yamasaki T, Izumi S, Murakami H, Mifuji K, Sawa M, Yasunaga Y, Adachi N, Ochi M (2017). Factors affecting the potential for posterior bony impingement after total hip arthroplasty. Bone Joint J.

[32] Shoji T, Yasunaga Y, Yamasaki T, Izumi S, Adachi N, Ochi M (2016). Anterior Inferior Iliac Spine Bone Morphology in Hip Dysplasia and Its Effect on Hip Range of Motion in Total Hip Arthroplasty. J Arthroplasty.

[33] Shoji T, Yasunaga Y, Yamasaki T, Mori R, Hamanishi M, Ochi MJIO (2013). Bony impingement depends on the bone morphology of the hip after total hip arthroplasty..

[34] Turley GA, Williams MA, Wellings RM, Griffin DR (2013). Evaluation of range of motion restriction within the hip joint. Med Biol Eng Comput.

[35] Weber M, Woerner M, Craiovan B, Voellner F, Worlicek M, Springorum HR, Grifka J, Renkawitz T (2016). Current standard rules of combined anteversion prevent prosthetic impingement but ignore osseous contact in total hip arthroplasty. Int Orthop.

[36] Widmer KH, Zurfluh B (2004). Compliant positioning of total hip components for optimal range of motion. J Orthop Res.

